# Enhanced Cellulase Production from *Bacillus subtilis* by Optimizing Physical Parameters for Bioethanol Production

**DOI:** 10.5402/2013/965310

**Published:** 2013-02-21

**Authors:** Deepmoni Deka, Saprativ P. Das, Naresh Sahoo, Debasish Das, Mohammad Jawed, Dinesh Goyal, Arun Goyal

**Affiliations:** ^1^Centre for the Environment, Indian Institute of Technology Guwahati, Guwahati, Assam 781039, India; ^2^Department of Biotechnology, Indian Institute of Technology Guwahati, Guwahati, Assam 781039, India; ^3^Department of Biotechnology and Environmental Sciences, Thapar University, Patiala, Punjab 147004, India

## Abstract

Effect of physical parameters such as initial pH, agitation (rpm), and temperature (°C) for cellulase production from *Bacillus subtilis* AS3 was investigated. Central composite design of experiments followed by multiple desirability function was applied for the optimization of cellulase activity and cell growth. The effect of the temperature and agitation was found to be significant among the three independent variables. The optimum levels of initial pH, temperature, and agitation for alkaline carboxymethylcellulase (CMCase) production predicted by the model were 7.2, 39°C, and 121 rpm, respectively. The CMCase activity with unoptimized physical parameters and previously optimized medium composition was 0.43 U/mL. The maximum activity (0.56 U/mL) and cell growth (2.01 mg/mL) predicted by the model were in consensus with values (0.57 U/mL, 2.1 mg/mL) obtained using optimized medium and optimal values of physical parameters. After optimization, 33% enhancement in CMCase activity (0.57 U/mL) was recorded. On scale-up of cellulase production process in bioreactor with all the optimized conditions, an activity of 0.75 U/mL was achieved. Consequently, the bacterial cellulase employed for bioethanol production expending (5%, w/v) NaOH-pretreated wild grass with *Zymomonas mobilis* yielded an utmost ethanol titre of 7.56 g/L and 11.65 g/L at shake flask and bioreactor level, respectively.

## 1. Introduction

Cellulases have versatile applications in textile, laundry, pulp and paper, fruit juice extraction, and animal feed additives [1]. In addition, they find use in saccharification of lignocellulosic agroresidues to fermentable sugars which can be used for production of bioethanol, lactic acid, and single-cell protein [2]. Bacteria have been widely explored for cellulase production owing to their high growth rate, expression of multienzyme complexes, stability at extreme temperature and pH, lesser feedback inhibition, and ability to withstand variety of environmental stress [1]. Among them, *Bacillus* sp. continues to be dominant bacterial workhorse due to the capacity to produce and secrete large quantities of extracellular enzymes [3, 4]. However, physical process parameters such as temperature, pH, and agitation speed play a vital role for the cellulase production efficiency of the microorganisms. Agitation speed is an important factor which governs the dissolved oxygen level in the culture broth that affects cell growth of cellulase producing microorganism [5]. However, higher agitation speed has been shown to inhibit cellulase activity [5, 6]. Analogous profile in growth and enzyme activity with change in pH and temperature is also a well-known fact [5, 7, 8]. Consequently, optimization of the culture conditions for improved enzyme production is essential.

The traditional “one-variable-at-a-time approach” for optimization disregards the complex interactions among various components. Statistically based experimental designs such as Placket-Burman design and response surface methodology (RSM) can be effectively used to study the effects of factors and to search for optimum levels of parameters for desired response [9]. Statistical design techniques have been successfully applied in many studies such as cellulose production by *Trichoderma reesei* [9], *Bacillus subtilis* AS3 [10], and xylanase production by *Bacillus pumilus* [11].

Simultaneous saccharification and fermentation (SSF) process combines enzymatic hydrolysis of cellulose with subsequent fermentation of reducing sugar (glucose) to ethanol [12]. SSF studies from lignocellulosic biomass such as wheat and rice straw, corn stalk, corn cobs, and forestry wastes using cellulase from natural sources [13, 14] have been reported. Owing to the inherent key enzymes for ethanol fermentation, alcohol dehydrogenase and pyruvate decarboxylase found in *Zymomonas mobilis,* research has been focused on it as a promising alternative ethanol producer for its high sugar uptake and improved ethanol tolerance [15].

In the present study, the physical process parameters such as initial pH, temperature, and agitation speed of the culture were optimized by central composite design technique using multiresponse analysis to enhance the alkaline CMCase activity from newly isolated *B. subtilis* (AS3). The optimal levels of physical process parameters predicted by the model were verified both in flask and bioreactor. Subsequently, the bacterial cellulase was employed for SSF trials on pretreated wild grass in shake flask and bioreactor with *Z. mobilis,* respectively. 

## 2. Materials and Methods

### 2.1. Reagents and Substrate

Carboxymethylcellulose (CMC) was purchased from Sigma-Aldrich (St. Louis, USA). All other chemicals and reagents of analytical grade like potassium dichromate (K_2_Cr_2_O_7_), glucose, yeast extract, and peptone used in the study were procured from Merck and Himedia laboratories (India). Lignocellulosic biomass wild grass (*Achnatherum hymenoides*) was provided by Professor Dinesh Goyal, Department of Biotechnology and Environmental Sciences, Thapar University, Patiala, Punjab, India. The biomass was washed thrice with water to remove adhering dust particles, dried at room temperature, and finally ground in a mixer grinder to 1 mm mesh size. 

### 2.2. Microorganisms and Cultivation Conditions

*Bacillus subtilis* AS3 (Genbank accession no. EU754025) isolated from cow dung and used for cellulase production was a kind gift from Professor D. Goyal, Thapar University, Patiala, Punjab, India. The culture was maintained in nutrient agar slant at 4°C and subcultured every 2 weeks. The inoculum was prepared by taking a loop full of culture from the nutrient agar slant in a 100 mL Erlenmeyer flask containing 25 mL of nutrient broth and incubated at 37°C and 180 rpm for 16–18 h (OD_600 nm_ = 0.6–0.8). 2% (v/v) of the fresh inoculum culture was added to 50 mL of optimized medium containing (g/L): CMC, 18; peptone, 8; yeast extract, 5; K_2_HPO_4_, 1; MgSO_4_·7H_2_O, 0.25; FeSO_4_·7H_2_O, 0.25; and MnCl_2_·4H_2_O, 0.5 [10] in 250 mL Erlenmeyer flask at different initial pH of the medium and incubated at different temperature and agitation as per the central composite experimental design presented in Table 1. Samples were collected at regular intervals of time for measurement of cell growth and CMCase activity.

*Zymomonas mobilis* (MTCC no. 2427) for fermentation was procured from Institute of Microbial Technology (IMTECH), Chandigarh, India. *Z. mobilis* was inoculated in autoclaved medium containing (g/100 mL) glucose, 2; yeast extract, 1, and KH_2_PO_4_, 0.2 with incubation at 30°C, 120 rpm. Aliquots measuring 1 mL from actively growing culture of *Z. mobilis* (2.1 × 10^6^ cells/mL) were transferred to 100 mL of fermentation medium. 

### 2.3. Optimization of Culture Conditions Using Response Surface Method (RSM)

In order to determine the best set of culture conditions to obtain maximum cellulase activity by *Bacillus subtilis* AS3, experiments were performed by varying the levels of culture conditions as per the central composite design (CCD). The culture conditions chosen for optimization study were pH, agitation speed (rpm), and temperature (°C); the total number of treatment combinations (experiments) was 20 = 2^*k*^ + 2*k* + *n*_0_, where “*k*” was the number of independent variables and “*n*_0_” the number of replicates performed at center point of the variables. Fourteen experiments were run with six replications at the center points to evaluate the pure error. Table 1 shows the range and levels of these three factors where the levels (−1, 0 and +1) of these culture conditions were chosen in such a way that the center point values (0) represented the factor levels mostly reported in the literature used for cellulase production. On the basis of the center point values, the low (−1) and high (+1) levels of the culture condition were determined in such a step change that the center point remains middle values of the low (−1) and high (+1) range of these factors. Furthermore, as per CCD to test all these factors in five ranges including coded value +*α* and −*α*, the uncoded values of these respective factors were calculated by solving the following equations:

(1) $$\begin{array}{c} x_{i}=\frac{X_{i}-X_{0}}{\Delta X_{i}},\;i=1,2,3,\ldots ,K, \end{array}$$

where *x*_*i*_ is the dimensionless value of an independent variable, *X*_*i*_ is the real value of an independent variable, *X*_0_ is the value of *X*_*i*_ at the center point, and Δ*X*_*i*_ is the step change, and where default alpha value for the 3 factor (*α* = 1.682) was chosen as per the CCD design. For fitting the experimental results by response surface regression procedure the following second order polynomial equation was used:

(2) $$\begin{array}{c} y=\beta_{o}+\sum_{i=1}^{k}\beta_{i}X_{i}+\sum_{i=1}^{k}\beta_{i}X_{i}^{\text{2}}+\sum_{i}\sum_{j}\beta_{ij}X_{i}X_{j}, \end{array}$$

where *y* is the predicted response, *k* is the number of factor variables, *X*_*i*_ and *X*_*j*_ are independent variables, *β*_0_ is the offset term, *β*_*i*_ is the *i*th linear coefficient, *β*_*ii*_ is the *i*th quadratic coefficient, and *β*_*ij*_ is the *ij*th interaction coefficient. The statistical software package MINITAB (Release 15.1, PA, USA) was used for regression analysis of the experimental data.

### 2.4. Multiple Response Optimization

Multiple response or desirability function is an analysis in which a number of responses (output variables) are measured simultaneously for each setting of a group of parameters (input variables) and is also called multiresponse analysis [16]. In systems having a large number of input variables and responses, the single response analysis has serious limitations as the optimum conditions for one response may not be suitable or practical for other responses and thus the meaning of optimum becomes unrealistic. The optimal conditions evaluated by this analysis are sometimes called near optimal for all responses. The optimization methodology based on the individual desirability using a desirability function evaluates how the settings optimize a single response. Optimal settings for input variables were determined by maximizing the composite desirability. These values are combined to determine the composite or overall desirability of the multiresponse system. An optimal point was where composite desirability reaches its maximum at 1. 

It was reported earlier that although higher cell growth was achieved at favorable agitation speed, pH, and temperature, at the higher biomass concentration, the cellulase activity is inhibited [5, 6, 8]. Similar observation was also obtained in the present study. Therefore, in order to optimize the cell growth for maximizing CMCase activity multiple response (desirability function) was applied by giving higher weight to enzyme activity as compared to cell growth. For this the following equation was used [16, 17]:

(3) $$\begin{array}{c} d_{i}\left({\hat{y}}_{i}\right)=\{\begin{array}{cc} 0, & \text{if}\;\;\hat{y}<L_{i},\\ {\left(\frac{{\hat{y}}_{i}-L_{i}}{T_{i}-L_{i}}\right)}^{r_{i}}, & \text{if}\;\;L_{i}\leq \hat{y}\leq T_{i},\\ 1, & \text{if}\;\;\hat{y}>T_{i}, \end{array} \end{array}$$

where $d_{i}({\hat{y}}_{i})$ is desirability function of a response and *L*_*i*_ and *T*_*i*_ are the lower and target values of response measured from experimental data. In the present study, while *L*_*i*_ for the two responses (CMCase activity and cell growth) were 0.104 U/mL and 1.65 mg/mL, respectively, *T*_*i*_ values were set at 0.56 U/mL and 2.0 mg/mL, respectively. ${\hat{y}}_{i}$ is the value of a response predicted by the second-order polynomial equations generalized before; *r*_*i*_ is the weight of desirability function of a response.

In this study, enzyme activity was given higher weight of 2 : 1 ratio as compared to cell growth. The overall desirability function (*D*) in turn was computed as shown below:

(4) $$\begin{array}{c} D={\left(\prod d_{i}^{wi}\right)}^{1/W}, \end{array}$$

where *d*_*i*_ is individual desirability for the *i*th response, *w*_*i*_ = importance of the *i*th response, and *W* = ∑*w*_*i*_. In the present study, *w*_*i*_ was taken at 2 : 1 ratio for enzyme activity and cell growth. For solving the desirability function, the statistical software package MINITAB (Release 15.1, PA, USA) was used.

### 2.5. Validation of the Experimental Model

In order to validate the model, experiments were performed in triplicate in a batch shake flask and 2 L stirred tank fermentor (Applicon, model Bio Console ADI 1025) using optimal levels of culture conditions (pH 7.2, 39°C, and 121 rpm) and optimized medium [10]. The laboratory scale bioreactor was operated at optimal levels of culture conditions and aeration rate of 1 vvm and 2% (v/v) inoculum. After 48 h, 1.0 mL of sample was withdrawn and absorbance at OD_600 nm_ was measured. The absorbance values were expressed as dry cell weight using a calibration curve. The samples were then centrifuged at 10,000 g for 10 min at 4°C and supernatant analyzed for enzyme activity. All measurements were carried out in triplicates and results' averages were taken as response.

### 2.6. Pretreatment of Wild Grass (*Achnatherum hymenoides*)

#### 2.6.1. Alkali Pretreatment

20 mL of 0.5 M NaOH was added to one gram of the powdered wild grass in a 250 mL Erlenmeyer flask [18]. Then, the mixture was autoclaved at 115°C at 15 psi for 10 min. Subsequently, the mixture was cooled to room temperature and treated with distilled water and 20 mM sodium phosphate buffer alternatively. Final wash was done with sodium phosphate buffer (20 mM, pH 6.0). Each ablution was followed by centrifugation (8,000 g, 10 min) till the pH became neutral and then the residues were dried in an oven at 70°C for 24 h.

#### 2.6.2. Acid Acetone Technique

One gram of substrate was incubated with 8 mL of concentrated phosphoric acid at 50°C at 120 rpm for one hour. The slurry was then poured in to 24 mL of chilled acetone and thoroughly mixed. The mixture was then centrifuged at 8,000 g for 10 minutes. The pellet was collected and centrifuged in distilled water for five minutes thrice. The pH was adjusted between 5 and 6 using NaOH during the third wash [19].

### 2.7. Simultaneous Saccharification and Fermentation (SSF) Experiments at Shake Flask and Bioreactor Level Using 1% (w/v) and 5% (w/v) Wild Grass (*A. hymenoides*)

One gram of the pretreated wild grass was taken in a 250 mL flask to which 100 mL of sodium phosphate buffer (pH 6.0, 20 mM) containing yeast extract (0.1%, w/v) and peptone (0.1%, w/v) was added. Then, 1 mL of isolated *B. subtilis* cellulase (3.3 U/mg, 0.5 mg/mL) along with 1 mL of *Z. mobilis* inoculum (2.1 × 10^6^ cells/mL) was added to the fermentation media. The fermentation was carried out at 120 rpm 30°C for three days and the sample was collected for every six hours with the monitoring of various parameters like cell OD_600 nm_, ethanol concentration (g/L), reducing sugar (g/L), and specific activity (U/mg). The SSF experiments were performed for alkali (NaOH) and acid-acetone-pretreated substrate. Batch SSF experiments were carried out in a 2 L lab scale fermenter (Applicon, model Bio Console ADI 1025) with a working volume of 1 L. 1% (w/v) of NaOH-treated wild grass along with l L of autoclaved fermentation media containing sodium phosphate buffer (pH 6.0, 20 mM) supplemented with yeast extract (0.1%, w/v) and peptone (0.1%, w/v) that was added in the fermenter. Subsequently, there was addition of 10 mL of *B. subtilis* cellulase (3.3 U/mg, 0.5 mg/mL) along with 10 mL of *Z. mobilis* inoculum (2.1 × 10^6^ cells/mL) into the SSF media. The bioethanol production was performed at 120 rpm, 30°C, and an aeration rate kept at 1 vvm which was controlled by a mass flow controller. The batch was run till 72 h with the sample collection at very 6 h interval. There was a constant monitoring of parameters like cell OD_600 nm_, ethanol concentration (g/L), reducing sugar (g/L), and specific activity (U/mg). Similar SSF procedure was followed on scaling up the NaOH-treated substrate concentration from 1% (w/v) to 5% (w/v) both at shake flask and reactor level. The fermentation conditions were also scaled up accordingly.

### 2.8. Analytical Methods

#### 2.8.1. Cell Growth Measurement

Cell growth was determined by measuring absorbance at optical density of 600 nm using a UV-visible spectrophotometer (Perkin Elmer, Model lambda-45) and the absorbance values were expressed as dry cell weight using a calibration curve of optical density (OD_600_) versus dry cell weight (g/L) of the sample. Dry cells weight of the centrifuged sample (10,000 g for 10 min) was measured by directly weighing the biomass after drying at 55°C to a constant weight.

#### 2.8.2. Assay of Enzyme Activity

The assay of cellulase was carried out in 100 *μ*L of reaction mixture containing 65 *μ*L of 2% (w/v) CMC in 50 mM sodium phosphate buffer (pH 6.0) and 35 *μ*L of cell-free supernatant and incubated at 45°C for 10 min. The CMCase activity was measured by estimating the liberated reducing sugar by the Nelson-Somogyi procedure [20, 21]. The reducing sugar was quantified from D-glucose standard curve. The absorbance was measured at 500 nm using a UV-visible spectrophotometer (Perkin Elmer, Model lambda-45) against a blank of 2% (w/v) CMC without enzyme. One unit (U) of CMCase activity is defined as the amount of enzyme that liberates 1 *μ*mole of reducing sugar (glucose) per min at 45°C in 50 mM sodium phosphate buffer, pH 6.0. 

#### 2.8.3. Ethanol Estimation

For ethanol content estimation, dichromate method was used where ethanol produced was converted to acid by reaction with dichromate [22]. The cell-free culture was diluted 10 times (reaction volume 10 mL) to which 2 mL of potassium dichromate (K_2_Cr_2_O_7_) (3.37 g/100 mL) was added and absorbance was measured on a spectrophotometer (Perkin Elmer, Model Lambda-45) at 600 nm.

## 3. Results and Discussion

### 3.1. Optimization of Culture Conditions Using RSM

For maximizing CMCase activity, the levels of the three important factors, pH, agitation speed (rpm), and temperature (°C), were varied using the central composite design of experiment. Table 1 represents the experimental and the model predicted values of CMCase activity along with cell growth, clearly depicting the close agreement of the experimental and predicted values with each other. The second-order response surface model outcomes were analyzed in the form of analysis of variance (ANOVA). Tables 2(a) and 2(b) present ANOVA of CMCase activity and cell growth profile of the culture, respectively. The Fisher's *F* value (21.18) for CMCase activity in the model owing to regression is found to be higher than the critical *F* value (*F*_0.05_ 9, 3 = 2.54) (Table 2(a)), indicating that most of the variations in the response could be explained by the regression model equation for CMCase activity. Generally, a large *F* value with a corresponding small *P* value indicates a high significance of the respective coefficient [23]. The associated *P* values are used to judge whether *F* was large enough to indicate statistical significance or not. The linear and square terms of both the regression models for CMCase activity and cell growth were found to be highly significant at *P* = 0.000. In the present study, the model *F* values of 21.18 (Table 2(a)) and 37.48 (Table 2(b)) for CMCase activity and cell growth, respectively, indicate that the respective regression models could explain most of the variation in the responses. These findings confirmed that the second-order polynomial models for CMCase activity and cell growth were adequate in predicting both the responses. These regression model equations are presented below:

(5) Y1=0.436816+0.071256X1+0.146789X2−0.073141X3 −0.005283X12+0.218983X22−0.020083X32 −0.068413X1X2+0.014319X1X3−0.123355X2X3,Y2=3.18098+0.21897X1+0.27103X2+0.50635X3 −0.86722X12−1.32722X22−0.53722X32 −0.02828X1X2+0.03536X1X3+0.33941X2X3,

where, *Y*_1_ = CMCase activity (U/mL), *Y*_2_ = cell growth (g/L), *X*_1_ is pH, *X*_2_ is temperature (°C), and *X*_3_ is agitation speed (rpm).

Further, to determine significance of the regression coefficients in the two models, the results were subjected to Student's *t*-test as presented in Table 3. From Table 3, it could be seen that the regression coefficients of linear and quadratic terms for all the factors in the models for CMCase activity and cell growth were found to be highly significant (*P* < 0.007); however, the quadratic coefficient due to pH and agitation speed for CMCase activity indicated insignificance on the responses (*P* > 0.4). From the Student's *t*-test of CMCase activity, the regression coefficient terms for interaction between temperature and agitation speed were found to be highly important (*P* < 0.009); however, interaction effects between pH and temperature revealed slightly less significance (*P* < 0.095). Other coefficient terms in the models did not seem to be have considerable significance (*P* > 0.7) on CMCase activity. In case of cell growth, the regression coefficient terms for interaction between temperature and agitation speed revealed some significance (*P* < 0.05) whereas no significant interaction was observed with other factors on cell growth of the culture. Such observations on significance of interaction effects between the variables would have been lost if the experiments were carried out by conventional methods [23].

**Figure 1:**
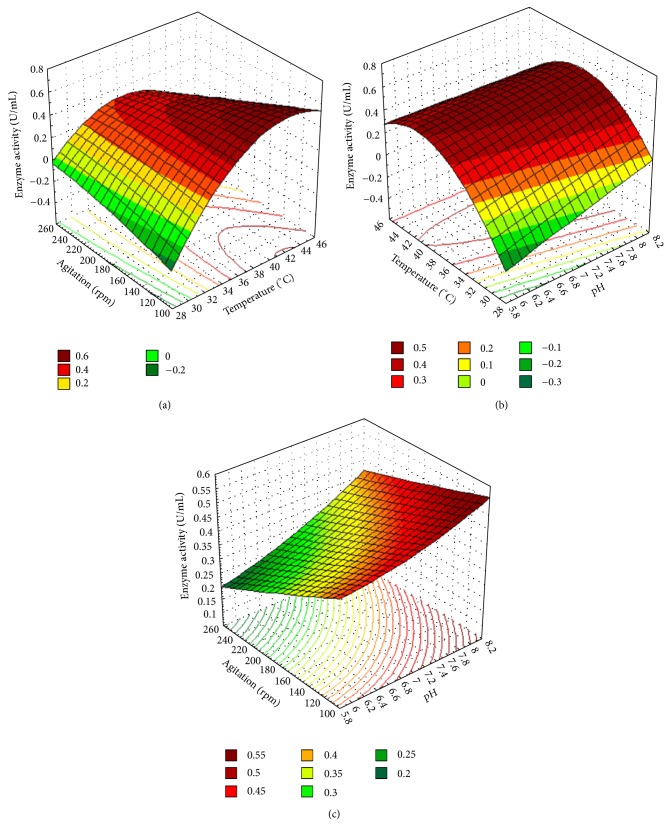
Three dimensional response surface plots for cellulase production showing the interaction effect between (a) temperature and agitation, (b) pH and temperature, and (c) pH and agitation.

In order to determine the optimal levels of the variables for maximum CMCase activity, three dimensional response surface plots as shown in Figure 1 were constructed by plotting the response against any two of the three independent variables and by maintaining the other variable at their middle (zero) levels.  Figure 1(a) representing the effects of temperature and agitation speed on CMCase activity at constant pH (7.0) demonstrated that although the enzyme activity was found sharply increasing with the temperature, beyond the agitation speed of 190 rpm, a sharp decline of the enzyme activity was observed indicating a strong negative interaction between the factors.  Figure 1(a) clearly revealed that higher agitation speed inhibited the enzyme activity.  Figure 1(b) signifying the interaction effect between pH and temperature on enzyme activity at constant agitation speed (180 rpm) showed that the enzyme activity stridently increased with increase of pH when temperature was lower than 40°C whereas reverse trend was observed with increase in temperature from 40–44°C revealing a negative interaction effect between these factors at their higher level.  Figure 1(c) displayed the effects of pH and agitation speed (rpm) on CMCase activity at constant temperature (37°C). The surface plot was found to be curvilinear clearly revealing no significant change in enzyme activity with change in the culture conditions and the optimum was observed near the central values of pH and agitation speed. 

To illustrate the above mentioned interaction effect between the variables in the study, typical contour plots between temperature and agitation speed and that between pH and temperature are depicted in bottom of the response surface plots of Figures 1(a) and 1(b). In general, the contours in such plots help in proper identification of the type of interactions between test variables; the surface confined in the smallest curve of such contour diagram can also be used to predict optimum response of the system. Hence, from the given plot in Figure 1(a), the corresponding coordinates in the region of the contour diagram gave the optimum values of the respective factors. Also, the response surface contour plots of mutual interaction between the variables temperature and agitation speed and that between pH and temperature, Figures 1(a) and 1(b), respectively, were found to be elliptical indicating significant interaction between these pairs of factors. Besides the two contour plots showing interaction between the variables, response surface contours drawn between pH and agitation speed in Figure 1(c) was circular indicating nonsignificant nature of their interactions.

Agitation speed is one of the important culture parameters that maintains homogenous conditions and disperses dissolved oxygen into smaller bubble thereby increasing the interfacial area and oxygen mass transfer rate for enhancing both substrate utilization and microbial activity [24]. The agitation speed was found to be optimum at 121 rpm. Other authors also reported similar optimum value of the parameter using *Bacillus* spp. [4, 25]. However, any further increase in the agitation speed more than 121 rpm did not improve the enzyme activity by the culture in the present study, which may be attributed to increased shear stress on the cells thus leading to reduced enzyme production [26]. Similar observations are also reported using *Bacillus amyloliquefaciens* [5], *Trichoderma reesei* [6], and *Thermomyces lanuginosus* [24], where cellulase production declines at higher agitation rates.

Temperature is also one of the most important parameters that influences enzyme activity and is essential for a fermentation process [25]. It was observed that when the culture temperature increased to an optimum level of 39°C an enhancement in CMCase activity was achieved. Similar observations on enhancement of cellulase activity were reported in other papers where optimum medium temperature for production of cellulase by *Bacillus subtilis* CY5 and *Bacillus circulans* were 40°C [27], and for *B. amyloliquefaciens* DL-3 [5] and *Bacillus pumilus* EB3 [28], it was 37°C which is, within the range, as obtained in the present study. However, temperature above and below the optimum level inhibited the cellulase activity by the microorganism probably due to inhibition of the multienzyme complex system of the cell [29]. At low temperature substrate transport across the cells is suppressed and lower product yields are attained [30]. Similarly, at higher temperature, the thermal denaturation of enzymes of the metabolic pathway could result in decreased enzyme production [30].

The pH of the growth medium influences many enzymatic reactions by affecting the transport of chemical products and enzymes across the cell membrane [31]. Our results also confirmed that medium pH is an important factor affecting cellulase activity. The optimum pH for maximum production of cellulase found in this study was 7.2. Similar finding was also reported by Ariffin et al. [28] and Rastogi et al. [4] for cellulase enzyme production. At the optimized physical parameters of pH 7.2, temperature 39°C, and agitation speed 121 rpm, the fermentation by *Bacillus subtilis* showed 33% enhancement in CMCase activity as compared to unoptimized parameters.

The maximum CMCase activity obtained using the optimized physical process parameters was 0.57 U/mL which was higher than many other reported values. For example, *Geobacillus* sp. and *Bacillus* sp. produced maximum CMCase activity of 0.074 U/mL and 0.12 U/mL, respectively, under optimized conditions [4, 25]. In another study, *Brevibacillus* sp. reported maximum cellulase activity of 0.02 U/mL under optimum culture conditions [4]. *Bacillus pumilus* EB3 and *Bacillus megaterium* recorded maximum cellulase activities of 0.076 U/mL and 0.102 U/mL, respectively, under optimized conditions in a 2 L stirred tank reactor [28, 32].

**Figure 2:**
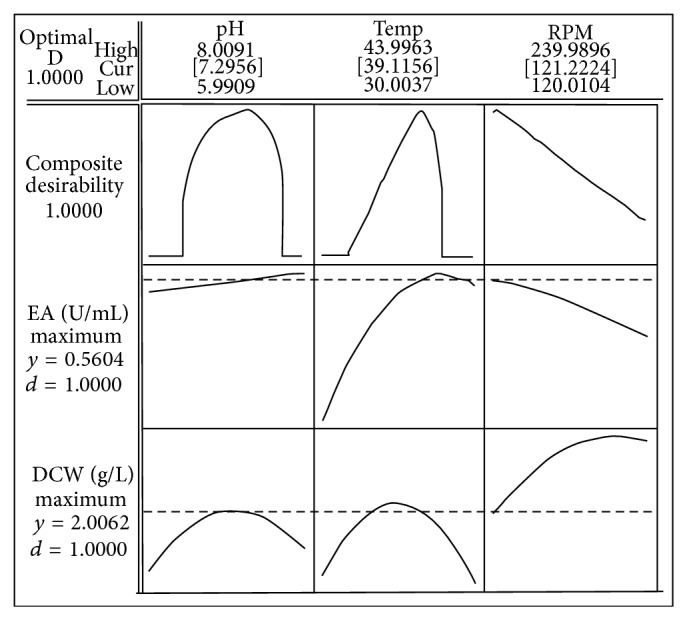
Desirability function plot showing the optimum level of physical process parameters.

In order to determine the optimal levels of each variable for maximizing CMCase activity, the method of desirability function was applied. The desirability function study in this multiple response optimization method shown in Figure 2 revealed that the overall desirability functions for CMCase activity and cell growth were close to 1 indicating the fact that the function increases linearly towards the desired target values of the two responses [16, 33]. In addition, individual desirability values of the two responses were calculated; while the value for cell growth was computed to be 1 with a maximum predicted response of 2.01 mg/mL, the value for CMCase activity was also found to be 1 with maximum predicted value of 0.56 U/mL. Thus, using the desirability function method for optimizing both the responses (discussed earlier), optimum values of the culture conditions were estimated to be pH 7.2, temperature 39°C, and agitation speed 121 rpm.

### 3.2. Validation of the Model

**Figure 3:**
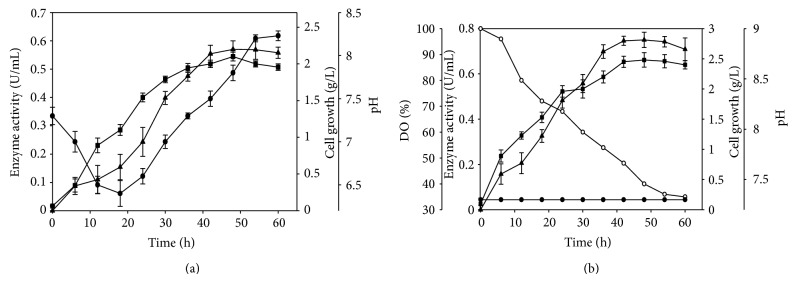
Cellulase production, cell growth, and pH profile of *B. subtilis* AS3 in (a) shake flask and in (b) fermentor containing optimized medium and physical parameters representing (▲) enzyme activity (U/mL), (■) cell growth (g/L), (●) pH, and (∘) dissolved oxygen (%) with time (h), respectively.

The CMCase activity was experimentally verified in batch shake flask and at 2 L stirred tank fermentor using optimized medium [10] and optimum values of physical parameters. The maximum CMCase activity and cell growth by *Bacillus subtilis* AS3 was 0.57 U/mL and 2.1 mg/mL in shake flask (Figure 3(a)) which are in very good agreement with the value predicted by the model (0.56 U/mL and 2.01 mg/mL). The enzyme activity with unoptimized physical parameters and optimized medium was 0.43 U/mL [10]. This showed 33% enhancement of CMCase activity after physical process parameter optimization (Table 4). The scale-up of batch cultivation from shake flask to bioreactor containing 1.0 L of the same optimized medium and optimized culture conditions yielded maximum CMCase activity of 0.75 U/mL (Figure 3(b)). A significant increase of 32% was observed due to controlled pH and maintenance of aeration in the fermentor throughout the cultivation which is not possible in shake flask (Table 4, Figure 3(b)). Shake flask experiments have limitations to control pH and dissolved oxygen level in the broth as compared to the fermentor. It was observed that in shake flask the pH of the culture medium showed variations with initial decline and then increasing trend in consensus with rise in enzyme activity at the end of cultivation (Figure 3(a)). The similar trends of pH variation have been observed with various other *Bacillus* strains [34]. pH control during fermentation was reported to be essential for increased cellulase production [34]. In scale up at 2 L bioreactor level, a maximum activity of 0.75 U/mL was observed with controlled pH at 7.2 after 48 h of fermentation (Figure 3(b)). The cell growth also showed a similar profile as cellulase production and reached its highest value at the late log phase (Figure 3(b)). The cell growth and CMCase activity data inferred the growth associated production of cellulase. A considerable increase in enzyme activity was observed in bioreactor as compared to flask level which may be due to the control of pH.

### 3.3. Pretreatment of Substrates and SSF at Shake Flask Level

The consequences of alkali (NaOH) and acid-acetone pretreatments were evaluated on wild grass (1%, w/v) subjected to simultaneous saccharification by *B. subtilis* cellulase and fermentation by *Z. mobilis.* These SSF trials were investigated to determine the outcome of various pretreatments on enzymatic hydrolysis and, in turn, the best ethanol titre (g/L). In case of alkali (NaOH) method, maximum ethanol concentration of 0.98 g/L from a reducing sugar content of 1.25 g/L with a yield coefficient of 0.098 (g of ethanol/g of substrate) was obtained (Table 5). With acid-acetone pretreatment, an ethanol concentration of 0.93 g/L was achieved for wild grass from reducing sugar (1.1 g/L) with a yield coefficient of 0.093 (g/g) (Table 5). 

On the basis of ethanol titre (g/L) obtained from SSF experiments involving two pretreatments, alkali (NaOH) treatment was found to be better over acid-acetone technique. Consequently, on increasing the wild grass concentration to 5% (w/v), an ethanol concentration of 7.56 g/L was achieved from a reducing sugar content of 9.08 g/L (Table 5). The ethanol yield was 0.15 (g/g). A 7.7-fold increment in ethanol titre was gained on increment of substrate concentration.

### 3.4. Fermenter Scale-Up Analysis for Bioethanol Production

**Figure 4:**
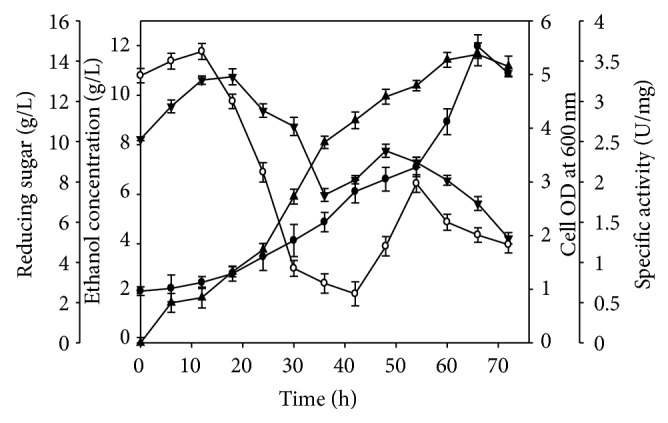
Simultaneous saccharification and fermentation (SSF) profile of wild grass (*A. hymenoides*) using *B. subtilis* AS3 cellulase and *Z. mobilis* in bioreactor (●) cell OD measured at 600 nm, (▲) ethanol concentration (g/L), (*▼*) reducing sugar (g/L), and (∘) specific activity (U/mg) with time, respectively. Cultivation conditions: working volume 1 L in 2 L bioreactor; initial pH 6.0; temperature 30°C; shaking 120 rpm; aeration rate 1 vvm incubation period 72 h; sampling interval every 6 h.

Owing to the controlled conditions of pH and aeration in batch reactor SSF, using 1% (w/v) wild grass with same enzyme and microbial combination, an ethanol titre of 2.23 g/L was obtained from a reducing sugar concentration of 2.5 g/L. The ethanol yield (g of ethanol/g of substrate) was 0.46 (Table 5). The SSF profile of 5% (w/v) wild grass involving *B. subtilis* cellulase and *Z. mobilis* showed an interesting relationship between cell growth, specific activity of the enzyme, rate of sugar utilization, and in turn rate of ethanol formation. As evident from Figure 4 there has been a sigmoidal increase in cell biomass after 24 h with a decrease after 66 h. During initial hours of fermentation there has not been a sharp increase in cell biomass owing to the acclimatization of the cells for further growth. The specific activity of the saccharifying cellulase was high during the early hours of SSF (18 h) (Figure 4) thereby releasing a maximum amount of utilizable sugars for the ready uptake of *Z. mobilis* for its growth and acclimatization. It is also observed that the specific activity of the enzyme decreased gradually during the late hours depicting an inhibition by the sugars released (30–42 h) (Figure 4). There was a gradual increase in the amount of reducing sugars (18 h) (13.22 g/L) (Table 5, Figure 4) with a substantial decrease after 30 h thereby depicting a sinusoidal behaviour between the rate of sugar utilization and ethanol formation. A gradual decline in sugar concentration after 48 h without any further rise in ethanol concentration indicated its utilization for maintenance and survival of the fermentative microbes. The ethanol formation in batch SSF at reactor level implicated a contrasting relationship between reducing sugar content and activity of bacterial cellulase. As observed from Figure 4, there was a sharp increment in ethanol formation from 24 h to 42 h after which a steady increase till 66 h (11.65 g/L) (Table 5, Figure 4) with a decline in the later stages of fermentation occured. Thus, on increasing the substrate concentration from 1% (w/v) to 5% (w/v), a 5.2-fold augmentation in ethanol titre (g/L) was witnessed on scaling up the SSF process at reactor level.

All these values of ethanol production are comparable with the other reported literature. An ethanol concentration of 0.09 g/L was obtained from 1% paper sludge waste using *Z. mobilis* [35]. An SSF experiment involving 30% solid content with commercial cellulase enzyme and *Z. mobilis* as fermentative organism gave an ethanol concentration of 60 g/L [15]. Reactor level aerobic batch fermentation with optimized process conditions offered a maximum ethanol concentration of 0.06 g/L from 1 g/L of pretreated sugarcane bagasse in [36].

## 4. Conclusions

The results demonstrated the effect of physical parameters such as initial pH, agitation (rpm), and temperature (°C) for cellulase production from *Bacillus subtilis* AS3. Central composite design of experiments followed by multiple desirability function was applied for the optimization of CMCase activity and cell growth. Among the three independent variables, the effect of the temperature and agitation was found to be significant. The maximum activity and cell growth predicted by the model were in consensus with values obtained using optimized medium and optimal values of physical parameters. After optimization, an enhancement in CMCase activity was recorded. On scale-up of cellulase production process with all the optimized conditions in bioreactor, an elevated activity was achieved. Consequently, the bacterial cellulase employed for bioethanol production expending pretreated wild grass with *Zymomonas mobilis* yielded a good ethanol titre at shake flask and bioreactor level, respectively. The present study, thus, clearly demonstrated, employing the statistical-based design technique, a significant enhancement in CMCase activity by *Bacillus subtilis* AS3 under optimized culture and medium conditions with subsequent usage in bioethanol production.

## Figures and Tables

**Table 1:** CCD showing experimental and regression model predicted CMCase activity (U/mL) and cell growth (g/L).

Run no.	pH	Temp (°C)	Agitation (rpm)	CMCase activity (U/mL)		Cell growth (g/L)	
				Measured	Predicted	Measured	Predicted
1	0 (7.0)	0 (37)	0 (180)	0.429 ± 0.05	0.437	3.16 ± 0.01	3.18
2	1 (7.6)	−1 (33)	1 (215)	0.311 ± 0.01	0.335	2.30 ± 0.05	2.39
3	−1 (6.4)	1 (41)	−1 (144)	0.499 ± 0.02	0.512	1.87 ± 0.12	1.85
4	*α* (8.0)	0 (37)	0 (180)	0.560 ± 0.01	0.503	2.74 ± 0.09	2.53
5	0 (7.0)	0 (37)	−*α* (120)	0.500 ± 0.08	0.490	2.08 ± 0.01	2.14
6	−*α* (5.9)	0 (37)	0 (180)	0.355 ± 0.05	0.360	1.98 ± 0.04	2.10
7	0 (7.0)	0 (37)	0 (180)	0.442 ± 0.02	0.437	3.23 ± 0.14	3.18
8	0 (7.0)	0 (37)	0 (180)	0.436 ± 0.03	0.437	3.19 ± 0.06	3.18
9	0 (7.0)	0 (37)	0 (180)	0.422 ± 0.06	0.437	3.16 ± 0.02	3.18
10	1 (7.6)	1 (41)	−1 (144)	0.532 ± 0.02	0.538	2.08 ± 0.08	2.06
11	1 (7.6)	1 (41)	1 (215)	0.322 ± 0.02	0.374	2.73 ± 0.05	2.93
12	0 (7.0)	0 (37)	0 (180)	0.448 ± 0.01	0.437	3.14 ± 0.04	3.18
13	−1 (6.4)	−1 (33)	−1 (144)	0.217 ± 0.07	0.202	1.88 ± 0.02	1.75
14	0 (7.0)	0 (37)	0 (180)	0.435 ± 0.04	0.437	3.19 ± 0.21	3.18
15	1 (7.6)	−1 (33)	−1 (144)	0.281 ± 0.11	0.325	1.87 ± 0.05	2.01
16	−1 (6.4)	1 (41)	1 (215)	0.334 ± 0.01	0.327	2.73 ± 0.02	2.67
17	0 (7.0)	0 (37)	*α* (240)	0.386 ± 0.05	0.344	3.30 ± 0.04	3.15
18	0 (7.0)	*α* (44)	0 (180)	0.385 ± 0.01	0.365	2.15 ± 0.18	2.13
19	0 (7.0)	−*α* (30)	0 (180)	0.103 ± 0.14	0.071	1.65 ± 0.05	1.58
20	−1 (6.4)	−1 (33)	1 (215)	0.161 ± 0.03	0.192	2.00 ± 0.01	2.08

Values are mean ± SE (*n* = 3).

**Table:** (a)

Source	df	SS	Adj MS	*F*	*P*	*R* ^2^
Regression	9	0.261363	0.029040	21.18	0.000	95.02
Linear	3	0.154385	0.051462	37.54	0.000	
Square	3	0.086877	0.028959	21.13	0.000	
Interaction	3	0.020102	0.006701	4.89	0.024	
Residual error	10	0.013708	0.001371			
Pure error	5	0.000404	0.000081			
Total	19	0.275071				

**Table:** (b)

Source	df	SS	Adj MS	*F*	*P*	*R* ^2^
Regression	9	6.30943	0.70105	37.48	0.000	97.12
Linear	3	1.82418	0.60806	32.51	0.000	
Square	3	4.36800	1.45600	77.85	0.000	
Interaction	3	0.11725	0.03908	2.09	0.165	
Residual error	10	0.18702	0.01870			
Pure error	5	0.00508	0.00102			
Total	19	6.49645				

df: degrees of freedom; SS: sum of squares; MS: mean sum of squares. *F*: Fisher's *F* value (calculated by dividing the MS owing to the model by that due to error); *P*: probability of incorrectly rejecting the null hypothesis when it is actually true.

**Table 3:** Result of Student's *t-*test for alkaline CMCase activity and the cell growth in the optimization study.

Term	CMCase activity (U/mL)		Cell growth (g/L)	
	*t*	*P*	*t*	*P*
Constant	28.927	0.000	57.032	0.000
pH (*X*_1_)	4.229	0.002	3.518	0.006
Temperature (°C) (*X*_2_)	8.712	0.000	4.355	0.001
Agitation (rpm) (*X*_3_)	−4.341	0.001	8.136	0.000
(*X*_1_^ 2 ^)	−0.192	0.852	−8.511	0.000
(*X*_2_^ 2^)	−7.938	0.000	−13.026	0.000
(*X*_3_^ 2^)	−0.728	0.483	−5.272	0.000
*X*_1_∗*X*_2_	−1.848	0.094	−0.207	0.840
*X*_1_∗*X*_3_	0.387	0.707	0.259	0.801
*X*_2_∗*X*_3_	−3.332	0.008	2.482	0.032

*t* statistic is the coefficient divided by its standard error, *P* is probability of incorrectly rejecting the null hypothesis when it is actually true.

**Table 4:** CMCase production at different levels of optimization.

Process conditions	Level of scale	CMCase activity (U/mL)	Enhanced production (fold)
Without any optimization	Shake flask	0.07 ± 0.02	—
With only optimised medium	Shake flask	0.43 ± 0.04	6
With optimised medium + optimised physical parameters	Shake flask	0.57 ± 0.01	8
With optimised medium + optimised physical parameters	Bioreactor	0.75 ± 0.03	11

Values are mean ± SE (*n* = 3).

**Table 5:** Bioethanol production by SSF from wild grass employing *B. subtilis* cellulase and *Z. mobilis*.

Pretreatment	Substrate concentration (%, w/v)	Mode of SSF	SSF process parameters		
			Reducing sugar∗ (g/L)	Ethanol yield (g/g)	Ethanol titre∗ (g/L)
Acid acetone	1%	Shake flask	1.10 ± 0.07	0.093	0.93 ± 0.07
Alkali	1%	Shake flask	1.25 ± 0.04	0.098	0.98 ± 0.06
Alkali	1%	Bioreactor	2.51 ± 0.03	0.22	2.23 ± 0.08
Alkali	5%	Shake flask	9.08 ± 0.06	0.15	7.56 ± 0.05
Alkali	5%	Bioreactor	13.22 ± 0.04	0.23	11.65 ± 0.04

^*^The values correspond to the maximum reducing sugar and maximum ethanol titre at a particular time.

Values are mean ± SE (*n* = 3).
